# Complement C3a Enhances the Phagocytic Activity of B Cells Through C3aR in a Fish

**DOI:** 10.3389/fimmu.2022.873982

**Published:** 2022-03-21

**Authors:** Zi-You Ma, Jia-Xin Liang, Wen-Shuo Li, Yuan Sun, Chang-Song Wu, Ya-Zhen Hu, Jun Li, Yong-An Zhang, Xu-Jie Zhang

**Affiliations:** ^1^ State Key Laboratory of Agricultural Microbiology, College of Fisheries, Huazhong Agricultural University, Wuhan, China; ^2^ Guangdong Provincial Key Laboratory of Pathogenic Biology and Epidemiology for Aquatic Economic Animals, Zhanjiang, China; ^3^ Engineering Research Center of Green Development for Conventional Aquatic Biological Industry in the Yangtze River Economic Belt, Ministry of Education, Wuhan, China; ^4^ Hubei Hongshan Laboratory, Huazhong Agricultural University, Wuhan, China; ^5^ School of Biological Sciences, Lake Superior State University, Sault Ste. Marie MI, United States

**Keywords:** C3a, C3aR, B cell, phagocytic activity, grass carp

## Abstract

The complement system is an important part of the immune system of teleost fish. Besides, teleost B cells possess both phagocytic activity and adaptive humoral immune function, unlike mammalian B1 cells with phagocytic activity and B2 cells specific to adaptive humoral immunity. However, the cross talk between complement system and phagocytic B cells in teleost fish still requires elucidation. Here, we show that, unlike tetrapods with a single *C3* gene, nine *C3* genes were identified from the grass carp (*Ctenopharyngodon idella*) genome, named *C3.1*-*C3.9*. Expression analysis revealed that C3.1 is the dominant C3 molecule in grass carp, for its expression was significantly higher than that of the other C3 molecules both at the mRNA and protein levels. The C3a fragment of C3.1 (C3a.1) was determined after the conserved C3 convertase cleavage site. Structural analysis revealed that C3a.1 consists of four α-helixes, with the C-terminal region forming a long α-helix, which is the potential functional region. Interestingly, we found that the recombinant GST-C3a.1 protein and the C-terminal α-helix peptide of C3a.1 both could significantly enhance the phagocytic activity of IgM^+^ B cells. Further study revealed that the C3a receptor (C3aR) was highly expressed in grass carp IgM^+^ B cells, and the phagocytosis-stimulating activity of C3a.1 could be dramatically inhibited by the anti-C3aR antibodies, indicating that C3a.1 performed the stimulating function through C3aR on IgM^+^ B cells. Taken together, our study not only uncovered the novel phagocytosis-stimulating activity of C3a, but also increased our knowledge of the cross talk between complement system and phagocytic B cells in teleost fish.

## Introduction

Like that in mammals, the complement system also plays crucial roles in the innate immune defense of teleost fish (1). The complement system of teleost fish is constituted of more than 30 components, among which complement C3 is a key molecule whose activation is essential for the function of the system (2, 3). After activation, C3 is cleaved into C3a and C3b by C3 convertase (4). In mammals, numerous studies have shown that C3a plays multiple immunoregulatory roles by interacting with the specific C3a receptor (C3aR) on immune cells, including the stimulation of respiratory burst in eosinophils, neutrophils, and macrophages (5–7), chemotaxis of eosinophils and mast cells (8, 9), induction of interleukin (IL)-1 production in monocytes (10), and modulation of dendritic cell activation (11). However, studies on teleost C3a are limited. Until now, we only know that teleost C3a can stimulate the respiratory burst in leukocytes but cannot chemoattract leukocytes (4, 12). Therefore, the immune functions of teleost C3a remain to be fully uncovered. Interestingly, unlike tetrapods with a single C3, all teleost fish possess multiple C3, e.g., zebrafish (*Danio rerio*) has eight C3 (13), while common carp (*Cyprinus carpio*) has five C3 (14). As a result, teleost fish can generate multiple C3a that are functionally active (4, 12).

In vertebrates, phagocytosis of leukocytes plays important roles in the immune defense of pathogens, as well as in the initiation of adaptive immunity (15, 16). Interestingly, in mammals, B2 cells are the main type of B cells specific to adaptive humoral immunity, while B1 cells are innate-like B cells with potent phagocytic activity (17, 18). However, unlike the differentiated mammalian B cells, teleost B cells possess both adaptive humoral immune function and phagocytic activity (19, 20). Moreover, after phagocytosis, teleost B cells can act as initiating antigen-presenting cells (APCs) to prime naive CD4^+^ T cell activation (21). Thereby, B cells act as an important bridge linking innate and adaptive immunity in teleost fish. Interestingly, we found that cathelicidin antimicrobial peptides can enhance the phagocytic activity of rainbow trout (*Oncorhynchus mykiss*) IgM^+^ and IgT^+^ B cells (22). Moreover, several studies have revealed that some cytokines can also enhance the phagocytic activity of teleost IgM^+^ B cells, including IL-10 (23), chemokine CK9 (24), and type I interferon-3 (25). These results indicated that the phagocytic activity of B cells can be modulated by multiple molecules to enhance the immunity of teleost fish.

The C3aR is a seven-transmembrane G protein-coupled receptor which has been widely characterized in mammals (26). In human, C3aR is widely expressed on various types of peripheral blood leukocytes (PBLs) but not B cells, including eosinophils, basophils, monocytes, and neutrophils (with the expression level from high to low), suggesting that eosinophils and basophils are the primary effector cells of C3a in the human peripheral blood (27). In teleost fish, the expression of C3aR on leukocytes has only been studied in rainbow trout, and the results showed that 83% of all PBLs express C3aR. However, significantly different from that in human, rainbow trout C3aR is highly expressed on all IgM^+^ B cells and, to a lesser extent, all granulocytes (26). Considering that teleost B cells possess potent phagocytic activity and express high levels of C3aR, and that a serum fraction containing C3a, C4a, and C5a can greatly enhance the phagocytic activity of rainbow trout (*Oncorhynchus mykiss*) head kidney leukocytes (HKLs) (28), it is reasonable to speculate that C3a can stimulate the phagocytic activity of teleost B cells through C3aR. To verify this hypothesis, we purified the GST tagged C3a fragment (GST-C3a.1) of grass carp (*Ctenopharyngodon idella*) C3.1 (the most abundant C3 molecule in grass carp), synthesized the C-terminal peptide of C3a.1 (C3a.1-CP), and found that GST-C3a.1 and C3a.1-CP both could significantly enhance the phagocytic activity of IgM^+^ B cells. Moreover, the phagocytosis-stimulating activity of GST-C3a.1 and C3a.1-CP could be dramatically inhibited by the anti-C3aR antibodies (Abs), indicating that C3a performed the B cell-stimulating function through C3aR. These results not only increased our knowledge of the cross talk between complement system and phagocytic B cells in teleost fish, but also inspired us to further study the application potential of C3a as an immunostimulant or as a molecular adjuvant by targeting B cells.

## Materials and Methods

### Experimental Fish

Healthy grass carp weighing 200 ± 20 g were purchased from Huangpi Hatchery (Wuhan, China), then maintained and acclimated to the laboratory aquarium tanks with water control system for at least two weeks before experiments.

### Searching, Identification, and Localization of *C3* Genes in Grass Carp Genome

We have re-sequenced and assembled the high-quality genome of grass carp, which has been deposited under NCBI BioProjects with an accession number PRJNA745929. The Basic Local Alignment Search Tool (BLAST) was used to search the *C3* genes in the grass carp genome using the published fish *C3* genes, especially zebrafish (*Danio rerio*) (13) *C3* genes. Interestingly, nine *C3* genes, named *C3.1*-*C3.9*, were found in grass carp. The reliability of the genes was confirmed by phylogenetic analysis using the neighbor-joining method bootstrapped 1000 times with the MEGA program (version 6.06). The grass carp *C3.1*-*C3.9* genes were deposited in the GenBank database (https://www.ncbi.nlm.nih.gov/genbank/) under accession numbers OL444976-OL444984, respectively. Synteny analysis of *C3* genes in grass carp, zebrafish, African clawed frog (*Xenopus laevis*), chicken (*Gallus gallus*), mouse (*Mus musculus*), and human (*Homo sapiens*) were conducted using the BLAST.

### Detection of the mRNA Expression of *C3* Genes in Grass Carp Tissues

To detect the mRNA expression of *C3* genes in grass carp tissues, healthy fish were firstly anaesthetized with tricaine methanesulfonate (MS-222; Sigma), then the blood was extracted from the caudal vein, and finally the remaining blood in the body was removed by cardiac perfusion using phosphate buffered saline (PBS; pH 7.4; Gibco). The tissues from liver, spleen, head kidney, gill, skin, and gut were sampled, and the total RNA was extracted using TRIzol reagent (Takara) according to the manufacturer’s instructions. The RNA was reverse transcribed into cDNA using the PrimeScript™ RT reagent Kit with gDNA Eraser (Takara). Quantitative real-time PCR (qPCR) was performed to analyze the mRNA expression levels of *C3* genes using FastStart Essential DNA Green Master Reagents (Vazyme) in a CFX Connect™ Real-Time System (Bio-Rad). The primers used are listed in **Supplementary Table 1**, and the amplification efficiencies of all the primer pairs were between 90 and 110%, calculated by using 10-fold series dilution of cDNA in qPCR. The specificity of the primer pairs was verified by the dissociation curves and sequencing the qPCR products (data not shown). The expression levels of *C3* genes were determined by the cycle threshold (Ct) method and normalized against the internal control *β-actin* using the 2^−ΔCt^ method.

### Determination of the C3a Fragment from Grass Carp C3.1

The amino acid sequence of grass carp C3.1 was aligned with human, mouse, and chicken C3 using the Clustal program (https://www.ebi.ac.uk/Tools/msa/clustalo/). Thereafter, the C3a fragment from grass carp C3.1 was determined after the conserved processing site of C3 convertase (data not shown). The tertiary structure of grass carp C3a.1 was modeled using the I-TASSER On-line Server (https://zhanggroup.org/I-TASSER/) based on the structure of human C3a (Protein Data Bank (PDB) code 4HW5). The structures of human C3a and grass carp C3a.1 were displayed using Pymol software.

### Production of Polyclonal Antibodies Against Grass Carp C3a.1

The peptide (VDGQECAKVFLHCCNEIKTRKNMKTEEEEMILAR) covering the C-terminal α-helix of grass carp C3a.1 (C3a.1-CP) was synthesized by GenScript Ltd. and analyzed by high-performance liquid chromatography (HPLC) and matrix-assisted laser desorption/ionization time-of-flight mass spectrometry (MALDI-TOF MS) to confirm that the purity was >95%. The specific pAbs against grass carp C3a.1 were produced by AtaGenix Ltd. using the C3a.1-CP as the antigen. Briefly, the C3a.1-CP was coupled to keyhole limpet hemocyanin (KLH) using the 1-ethyl-3-(3-dimethylamino) propyl carbodiimide (EDC)/*N*-hydroxysuccinimide (NHS) coupling method, then KLH-C3a.1-CP was used to raise pAbs in rabbits. The rabbit IgG in the antiserum was first purified using a HiTrap protein G column, then the grass carp C3a.1 specific pAbs were purified by affinity chromatography using the C3a.1-CP coupled cyanogen bromide (CNBr)-activated Sepharose.

### Detection of the C3.1 Proteins in Grass Carp Serum

The grass carp serum (2ml) was loaded onto a Superdex-200 FPLC column (GE Healthcare) in an ÄKTA purification system (GE Healthcare), then eluted with PBS (pH 7.4) to collect fractions (1 ml per fraction). The fraction with molecular masses of 180-200 kDa was collected to detect C3.1 by Western blot. Briefly, the fractions were resolved by 8% sodium dodecyl sulfate polyacrylamide gel electrophoresis (SDS-PAGE) under non-reducing conditions, and the gel was then stained with Coomassie blue dye, followed by transferring to a nitrocellulose blotting membrane (Pall). The membrane was blocked for 1 h at room temperature in TBST buffer (25 mM Tris˗HCl, 150 mM NaCl, 0.1% Tween 20, pH 7.5) containing 5% nonfat dry milk, then incubated with rabbit anti-grass carp C3a.1 pAbs at 4°C overnight. After three washes with TBST, the membrane was incubated with HRP-conjugated goat anti-rabbit IgG Abs (Thermo) for 2 h at room temperature. After three washes with TBST, the membrane was stained with ECL chemiluminescence substrate (Biosharp) and photographed by a chemiluminescence imager (Amersham Imager 680, GE Healthcare). The gel slice corresponding to the C3.1 band was subjected to in-gel tryptic digestion, followed by liquid chromatography tandem-mass spectrometry (LC-MS/MS) analysis.

### Fractionation and Detection of C3a.1 in Activated Grass Carp Serum

The grass carp serum was activated using a previously described method (29). Briefly, 1 ml grass carp serum were incubated with 80 μl rabbit red blood cells (RaRBC; SenBeiJia) and 1ml 20 mM magnesium sulfate heptahydrate (Sinopharm Chemical Reagent) at 20°C for 100 min. Then, RaRBC-activated serum was centrifuged at 400 × *g* for 10 min to collect the supernatant. The supernatant (2ml) was loaded onto a Superdex-200 FPLC column and eluted with PBS (pH 7.4) in an ÄKTA purification system as described above. The fractions with molecular masses of 8-9 kDa were collected to detect C3a.1 by Western blotting as above described using rabbit anti-grass carp C3a.1 pAbs.

### Expression and Purification of Glutathione S-Transferase (GST)-Tagged Recombinant C3a.1

The cDNA sequence encoding grass carp C3a.1 was amplified by PCR using the primer pair exC3a.1-F/exC3a.1-R listed in **Supplementary Table 1**. The PCR product was digested and inserted into pGEX-6P-1 using Xho I and BamH I restriction enzymes. The pGEX-C3a.1 plasmid was transformed into *Escherichia coli* Rosetta competent cells (YouBio), then the cells were cultured at 37°C for 12 h supplemented with 100 mg/L ampicillin. Thereafter, the cells were induced with 0.1 mM IPTG at 28°C for 6 h, followed by centrifugation at 5000 × *g* for 10 min. The cells were lysed by a high-pressure homogenizer (ATS) for 10 min, centrifuged at 10000 × *g* for 10 min, and then the supernatant was filtered and incubated with Glutathione Sepharose 4B resin (Amersham Biosciences) at 4°C for 6 h. The unbounded proteins were removed from the resin by adequate washing with PBS. The GST-C3a.1 fusion protein on the resin was eluted by 20 mM reduced glutathione at 4°C. The eluted GST-C3a.1 was finally dialyzed against PBS (pH 7.4).

### Isolation of Leukocytes

Grass carp were anesthetized and perfused as above described. The head kidney leukocytes (HKLs) were isolated with Percoll (GE Healthcare) using the method described in our previous studies (20, 30). Briefly, the head kidney was dissociated into cell suspensions in Dulbecco’s Modified Eagle Medium (DMEM; Invitrogen), then passed through a 100 μm nylon cell strainer (BD Biosciences). The cell suspensions were placed onto 34/51% discontinuous Percoll gradients, then centrifuged at 400 × *g* for 30 min at 4°C. The leukocytes at the 34/51% interface were collected and washed twice with PBS containing 2% fetal bovine serum (FBS, Gibco).

### Assay of Phagocytosis

The phagocytosis-stimulating activities of GST-C3a.1 and C3a.1-CP to grass carp IgM^+^ B cells were measured using the method described in our previous study with minor modifications (22). Briefly, HKLs in DMEM were seeded in 96-well plates (Nunc) (200 μl per well) at 1 × 10^6^ cells/well, then incubated with GST-C3a.1 or C3a.1-CP at different concentrations as well as fluorescent beads (Fluoresbrite Yellow Green Microspheres; 1.0 μm in diameter; Polysciences) at a cell/bead ratio of 1:20 at 28°C for 2 h. PBS and GST were used as the blank and negative controls, respectively, in the assay for GST-C3a.1, while PBS and the C-terminal peptide (VQQPDCREPFLSCCQFAESLRKKSRDKGQAGLQR) of human C4a (huC4a-CP) were used as the blank and negative controls, respectively, in the assay for C3a.1-CP. The huC4a-CP was synthesized by GL Biochem Ltd. and analyzed by HPLC and MALDI-TOF MS to confirm that the purity was >95%. After incubation, the non-ingested beads in cell suspensions were removed by centrifugation (400 × *g* for 10 min at 4°C) over a cushion of 3% (weight/volume) BSA (Thermo) in PBS supplemented with 4.5% (weight/volume) D-glucose (Sigma). Subsequently, the cells were washed and stained with mouse anti-grass carp IgM monoclonal Abs (mAbs; 1 μg/ml) (30), followed by staining with allophycocyanin (APC)-conjugated goat anti-mouse IgG Abs (2 μg/ml; BioLegend). Finally, the phagocytic activity of grass carp IgM^+^ B cells was detected using a flow cytometer (FACSVerse™, BD Biosciences) and the data were analyzed using FlowJo software (Tree Star).

### Production of pAbs Against Grass Carp C3aR

The rabbit anti-grass carp C3aR pAbs were raised and purified by AtaGenix Ltd. as above described using an antigenic peptide at the N-terminal of grass carp C3aR (NESHYNDDMNSSGYDC) (GenBank accession number: MG599686.1). To verify the specificity of the anti-C3aR pAbs, a blocking experiment to anti-C3aR pAbs was performed using the antigenic peptide as previously described (31). Briefly, the anti-C3aR pAbs were preincubated with the antigenic peptide at 1:0, 1:10, 1:50, 1:100, and 1:200 molar ratios for 30 min, then stained HKLs for flow cytometry. To further verify the specificity of the pAbs, HKLs were stained with rabbit anti-C3aR pAbs as described above, then the C3aR^+^ lymphocytes and C3aR^-^ lymphocytes were sorted by FACS. The mRNA expression of C3aR in the sorted cells were detected by qPCR as above described using the primers listed in **Supplementary Table 1**.

### Detection of the Expression of *C3aR* in Grass Carp IgM^+^ B Cells

To detect the mRNA expression of C3aR in IgM^+^ B cells, HKLs were isolated from grass carp and stained with mouse anti-grass carp IgM mAbs as described above. The IgM^+^ B cells, IgM^-^ lymphocytes (Lym), subset I myeloid cells (Mye I), and subset II myeloid cells (Mye II) were sorted using a fluorescence-activated cell sorter (FACS; FACSAria™ III, BD Biosciences). Total RNA was extracted from the sorted cells using a RNeasy Mini Kit (MAGEN) and the cDNA was synthesized using Hiscript III Reverse Transcriptase (TaKaRa). The expression of *C3aR* was detected by FastStart Essential DNA Green Master Reagents (Vazyme) in a Real-Time System (CFX Connect™, Bio-Rad). The primers used are listed in **Supplementary Table 1**. The expression level of *C3aR* was analyzed using the 2^−ΔCT^ method, with *β-actin* as the internal control.

To detect the protein expression of C3aR on IgM^+^ B cells, immunofluorescence microscopy was conducted using the method described in our previous study (32). Briefly, HKLs were spun onto microscopy slides using a Cytospin (Thermo), then fixed and blocked with PBS containing 2% BSA at room temperature for 1 h. Thereafter, the HKLs were co-stained with mouse anti-grass carp IgM mAbs (1 μg/ml) and rabbit anti-grass carp C3aR pAbs (1 μg/ml) overnight at 4°C. The mouse IgG isotype control Abs (1 μg/ml; BioLegend) and rabbit IgG isotype control Abs (1 μg/ml; BioLegend) were used as the negative controls. After washing with PBS, the HKLs were co-stained with Alex Flour 488-conjugated goat anti-mouse IgG (2 μg/ml; Jackson ImmunoResearch) and Cy5-conjugated goat anti-rabbit IgG (2 μg/ml; Jackson ImmunoResearch) Abs at room temperature for 1 h. After washing with PBS, the HKLs were stained with DAPI (1 μg/ml; Beyotime) at room temperature for 5 min. Finally, the HKLs were imaged with an inverted confocal microscope (Nikon N-STORM).

### Blocking C3aR by Anti-C3aR pAbs

To clarify the mechanism of the phagocytosis-stimulating activities of C3a.1 to grass carp IgM^+^ B cells, a blocking experiment to C3aR was performed using anti-C3aR pAbs. Briefly, HKLs in DMEM were seeded in 96-well plates (200 μl per well) at 1 × 10^6^ cells/well, then GST-C3a.1 (10 nM) or C3a.1-CP (10 nM), anti-C3aR pAbs or isotype control Abs, as well as fluorescent beads (cell: bead = 1: 20) were added. The cell suspensions were incubated at 28°C for 2 h. Thereafter, the non-ingested beads in cell suspensions were removed, then the cells were stained with mouse anti-grass carp IgM mAbs, and finally the phagocytic activity of grass carp IgM^+^ B cells was detected using a flow cytometer as above described.

### Statistical Analysis

The statistic *p* value was calculated by one-way ANOVA with a Dunnett *post hoc* test (SPSS Statistics, version 19, IBM). A *p* value < 0.05 was considered statistically significant.

## Results

### Nine *C3* Genes Exist in Grass Carp

**Figure 1:**
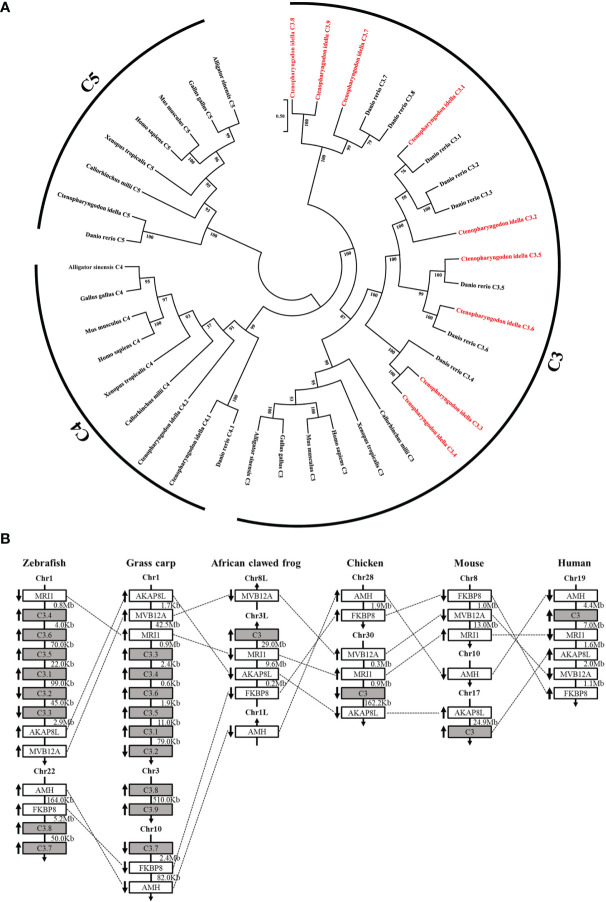
Relationship of grass carp *C3* genes with the known *C3*, *C4*, and *C5* genes from representative vertebrates. **(A)** Phylogenetic relationship of the nine C3 proteins from grass carp with the known C3, C4, and C5 from representative vertebrates. The phylogenetic tree based on protein sequences was constructed using the neighbor-joining method bootstrapped 1000 times with the MEGA program. Grass carp C3.1-C3.9 are shown in red. GenBank accession numbers of the used sequences are shown below: *Ctenopharyngodon idella* C3.1, OL444976; *C. idella* C3.2, OL444977; *C. idella* C3.3, OL444978; *C. idella* C3.4, OL444979; *C.. idella* C3.5, OL444980; *C. idella* C3.6, OL444981; *C. idella* C3.7, OL444982; *C. idella* C3.8, OL444983; *C. idella* C3.9, OL444984; *C. idella* C4.1, MG599708; *C.. idella* C4.2, MG599709; *C. idella* C5, MT150869; *Danio rerio* C3.1, NP_571317.1; *D. rerio* C3.2, NP_571318.1; *D. rerio* C3.3, NP_001032313.1; *D. rerio* C3.4, XP_002660623.2; *D. rerio* C3.5, XP_002660624.2; *D. rerio* C3.6, NP_001008582.3; *D. rerio* C3.7, NP_001093490.1; *D. rerio* C3.8, NP_001093483.1; *D. rerio* C4.1, XP_005157429.1; *D. rerio* C5, XP_001919226.4; *Homo sapiens* C3, EAW69070.1; *H. sapiens* C4, AAI44547.1; *H. sapiens* C5, NP_001726.2; *Mus musculus* C3, NP_033908.2; *M. musculus* C4, XP_006523594.1; *M. musculus* C5, NP_034536.3; *Gallus* C3, AAA64694.1; *G. gallus* C4, NP_001070701.1; *G. gallus* C5, XP_415405.5; *Callorhinchus milii* C3, XP_042200573.1; *C. milii* C4, XP_042200730.1; *C. milii* C5, XP_007900925.2; *Xenopus tropicalis* C3, XP_031755334.1; *X. tropicalis* C4, NP_001107156.1; *X. tropicalis* C5, NP_001190988.1; *Alligator sinensis* C3, XP_006023407.1; *A. sinensis* C4, XP_025048727.1; *A. sinensis* C5, XP_014382164.1; **(B)** Synteny analyses of grass carp *C3* genes with other vertebrate *C3* genes. *C3* genes are indicated by solid grey boxes. Arrows indicate gene transcription orientations. Chr, chromosome.

Nine *C3* genes were identified from the grass carp genome through homology searching, named *C3.1*-*C3.9* (**Figure 1**). Phylogenetic analysis showed that the nine C3 proteins in grass carp were closely clustered with the eight C3 in zebrafish (**Figure 1A**). The nine *C3* genes are located in three different chromosomes (chr) in grass carp, with *C3.1*-*C3.6* in chr1, *C3.7* in chr10, while *C3.8*-*C3.9* in chr3. Among these grass carp *C3* genes, *C3.1*-*C3.6* showed more conserved synteny with those *C3* genes in tetrapods (**Figure 1B**).

### C3.1 is the Dominant C3 Molecule in Grass Carp

**Figure 2:**
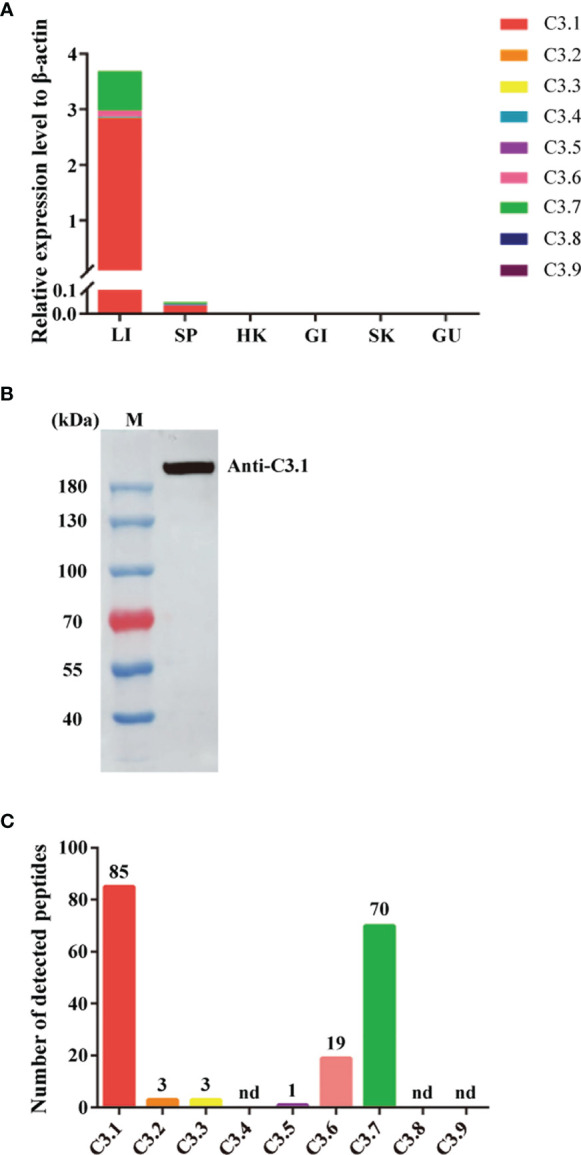
Expression patterns of the nine *C3* genes of grass carp at mRNA and protein levels. **(A)** Tissue expression patterns of the nine *C3* genes of grass carp at the mRNA level. The expression levels were analyzed by qPCR and normalized against the expression of *β-actin* using the 2^−ΔCt^ method. Abbreviations for tissues are as follows: LI, liver; SP, spleen; HK, head kidney; GI, gill; SK, skin; GU, gut. Data present the mean value of 5 fish. **(B)** Detection of C3.1 in grass carp serum by Western blotting. The grass carp serum was separated by gel filtration chromatography, and the fraction with molecular masses of 180-200 kDa was collected to detect C3.1 by Western blotting. M, marker (Thermo). **(C)** Expression patterns of the nine C3 molecules in grass carp serum. The gel slice corresponding to the C3.1 band in **(B)** was subjected to in-gel tryptic digestion, followed by LC-MS/MS analysis. The data shown are the copy numbers of the detected peptides from grass carp C3 molecules. nd, not detected.

The mRNA expression levels of the grass carp *C3* genes were analyzed by qPCR in various tissues (**Figure 2A**). As shown, the *C3* genes were mainly expressed in liver, though their expression also could be detected in the other tissues tested. Among the grass carp *C3* genes, the expression level of *C3.1* was significantly higher than that of the other *C3* genes, with that of *C3.7* being the second one. Moreover, the protein expression levels of the grass carp *C3* genes were analyzed by LC-MS/MS in serum (**Figure 2B, C**). The results also showed that C3.1 is the most abundant C3 molecule in grass carp serum (**Figure 2C**). All these results indicated that C3.1 is the dominant C3 molecule in grass carp.

### Native C3a.1 is Generated in the Activated Grass Carp Serum

**Figure 3:**
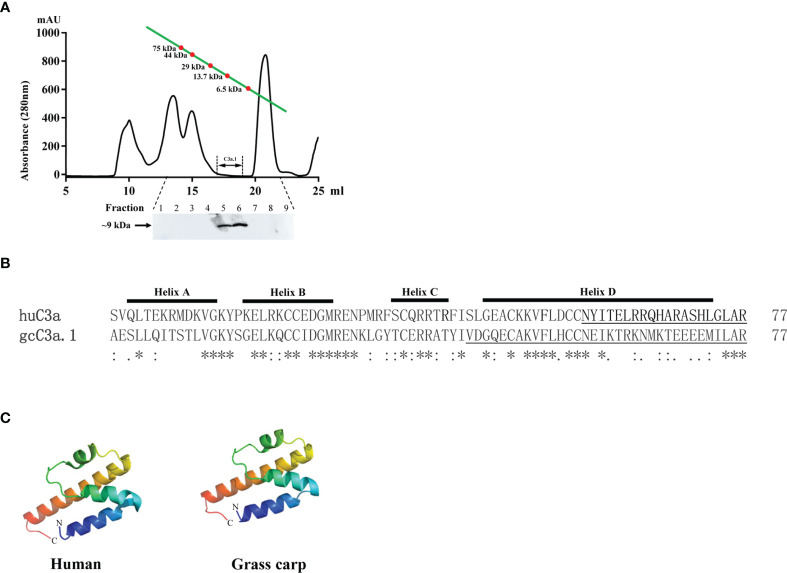
The amino acid sequence and tertiary structure of grass carp C3a.1. **(A)** Native C3a.1 is generated in the complement-activated grass carp serum. The. complement-activated grass carp serum was separated by gel filtration chromatography, and the fractions with molecular masses of 8-9 kDa were collected to detect C3a.1 by Western blotting. **(B)** Amino acid sequence alignment of grass carp (gc) C3a.1 with human (hu) C3a. The sequence alignment was conducted using the Clustal program. The functional region of huC3a as well as the C-terminal peptide of gcC3a.1 synthesized for functional study are underlined. **(C)** Tertiary structure of grass carp C3a.1. The tertiary structure of grass carp C3a.1 was modeled using the I-TASSER On-line Server based on the structure of human C3a. The. symbols *, : , and . indicate the identities of the corresponding amino acid residues, from high to low.

To confirm the existence of native C3a.1 in grass carp serum, we used the rabbit anti-grass carp C3a.1 pAbs to detect C3a.1 with Western blotting in RaRBC-activated serum fractionated by gel filtration chromatography. A band of ~9 kDa, which was consistent with the predicted molecular mass of grass carp C3a.1 (8.8 kDa), was immunoreactive with the anti-grass carp C3a.1 pAbs (**Figure 3A**). This result indicated that native C3a.1 is generated in the grass carp serum after the activation of the complement system.

### C3a.1 Enhances the Phagocytic Activity of Grass Carp IgM^+^ B Cells

**Figure 4:**
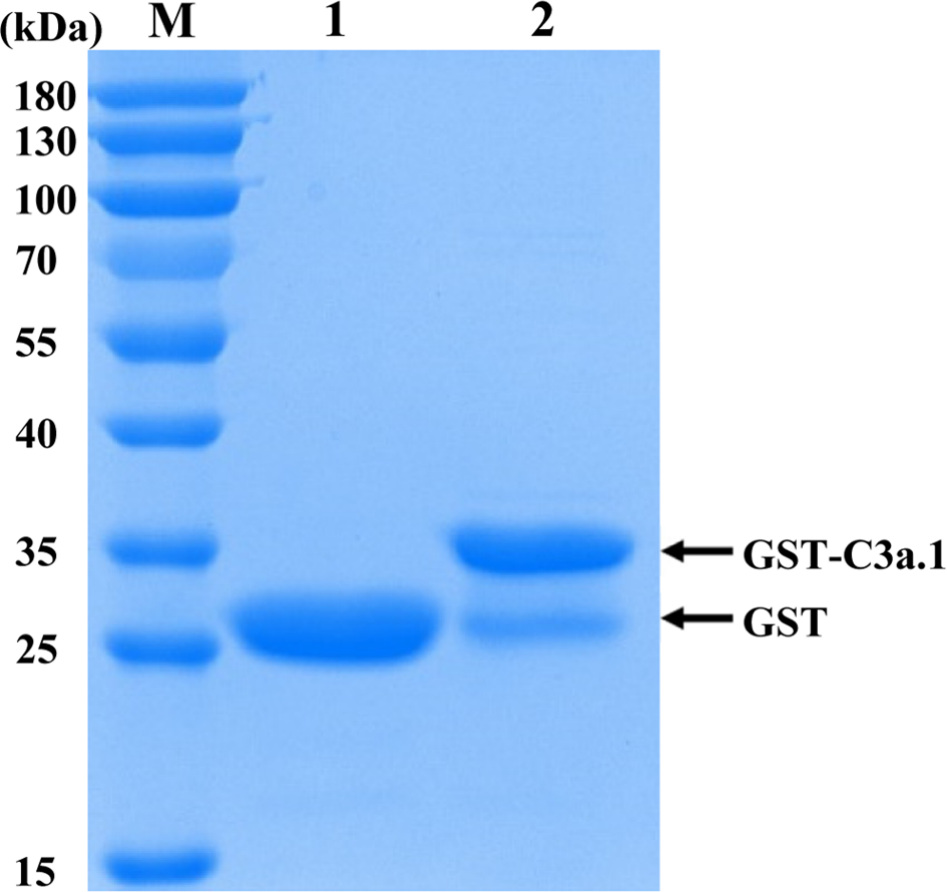
SDS-PAGE analysis of the purified GST and GST-C3a.1 proteins. M, marker (Thermo); lane 1, purified GST protein; lane 2, purified GST-C3a.1 protein.

**Figure 5:**
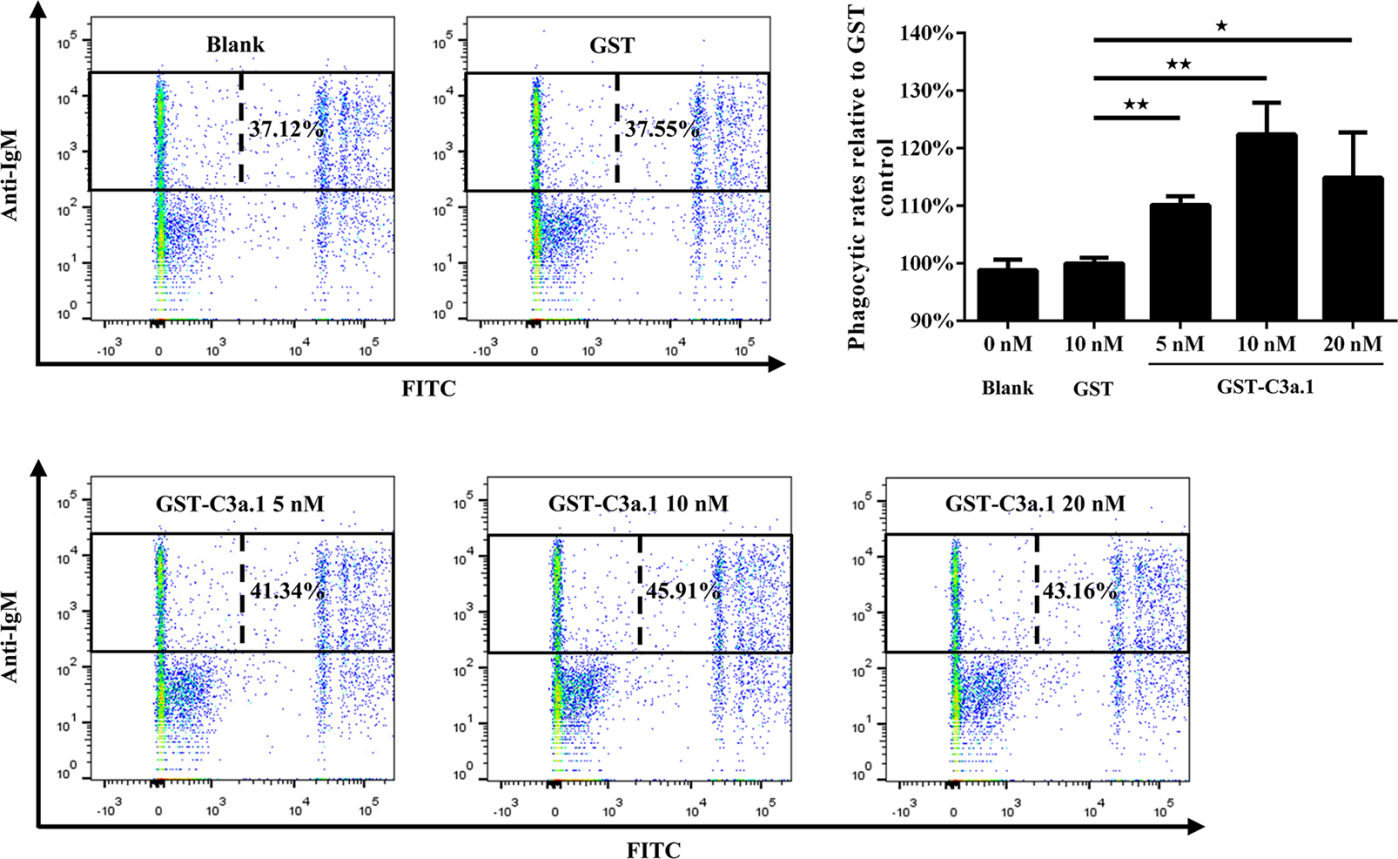
C3a.1 enhances the phagocytic activity of grass carp IgM^+^ B cells. HKLs were incubated with GST-C3a.1 and fluorescent beads at 28°C for 2 h. The PBS and GST were used as the blank and negative controls, respectively. After incubation, the non-ingested beads in cell suspensions were removed, and the cells were stained with mouse anti-grass carp IgM mAbs. Finally, the phagocytic activity of grass carp IgM^+^ B cells was detected using flow cytometry. One representative result was shown in the dot plot of flow cytometry. Data are presented as mean ± SEM (n = 3 fish), and the statistic *p* value was calculated by one-way ANOVA with a Dunnett *post hoc* test (*p < 0.05, **p < 0.01).

The SDS-PAGE analysis showed that high purity GST-C3a.1 fusion protein was successfully expressed and purified from *E. coli* (**Figure 4**). To determine if C3a.1 could increase the phagocytic activity of grass carp IgM^+^ B cells, a phagocytosis assay was conducted using flow cytometry. As shown in **Figure 5**, compared with the GST control, GST-C3a.1 significantly enhanced the phagocytic activity of grass carp IgM^+^ B cells in a concentration-dependent manner. The optimal phagocytosis-stimulating concentration of GST-C3a.1 is about 10 nM, with the phagocytic activity of grass carp IgM^+^ B cells being increased by more than 20%. Notably, the GST tag did not influence the phagocytic activity of grass carp IgM^+^ B cells as compared with the blank control, indicating that the phagocytosis-stimulating activity of C3a.1 is specific.

### C3a.1 Enhances the Phagocytic Activity of Grass Carp IgM^+^ B Cells Through the C-Terminal α-helix Region

**Figure 6:**
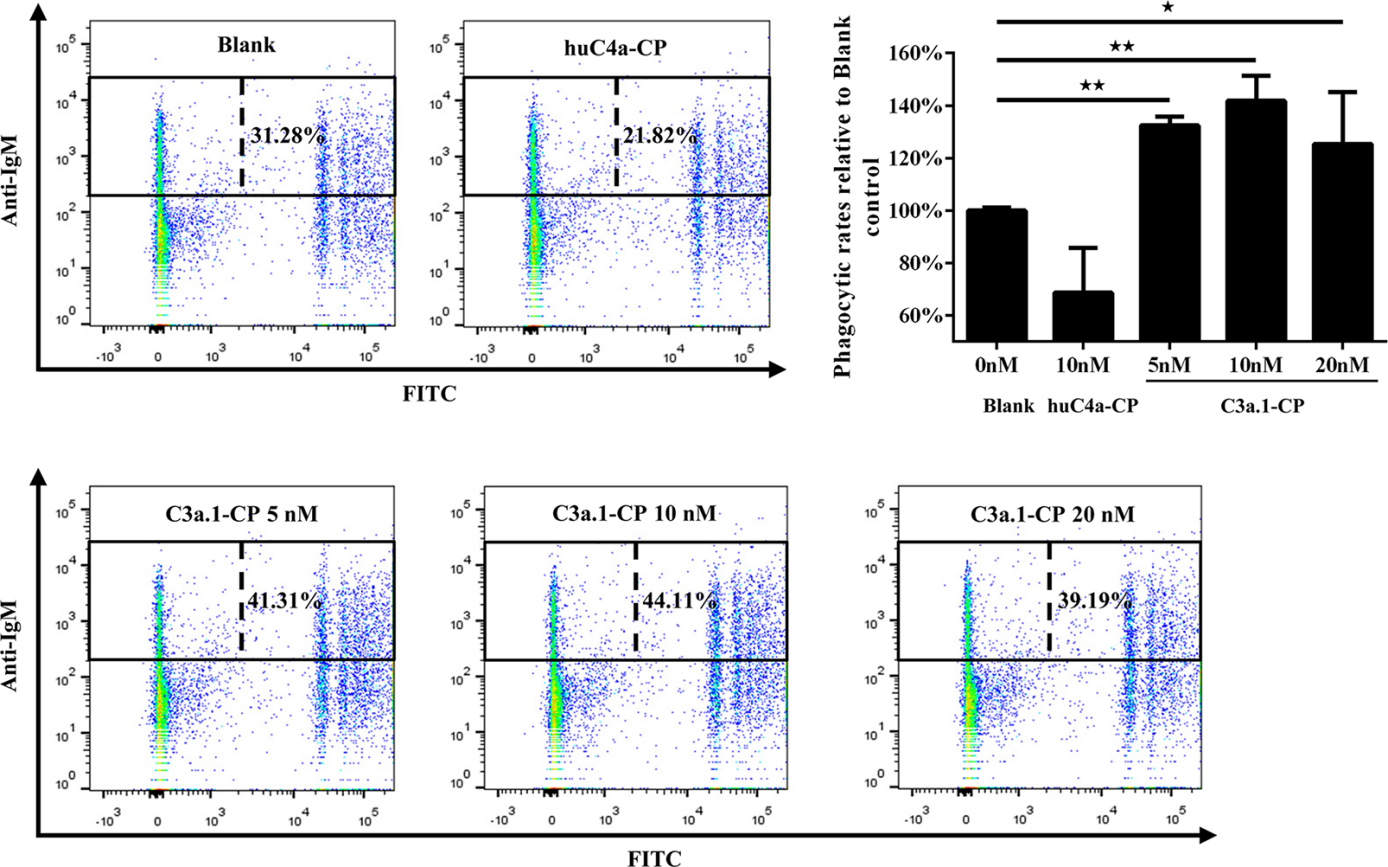
The C-terminal α-helix peptide of C3a.1 (C3a.1-CP) enhances the phagocytic activity of grass carp IgM^+^ B cells. HKLs were incubated with C3a.1-CP and fluorescent beads at 28°C for 2 h. The PBS and C-terminal peptide of human C4a (huC4a-CP) were used as the blank and negative controls, respectively. After incubation, the non-ingested beads in cell suspensions were removed, and the cells were stained with mouse anti-grass carp IgM mAbs. Finally, the phagocytic activity of grass carp IgM^+^ B cells was detected using flow cytometry. One representative result was shown in the dot plot of flow cytometry. Data are presented as mean ± SEM (n = 3 fish), and the statistic *p* value was calculated by one-way ANOVA with a Dunnett *post hoc* test (*p < 0.05, **p < 0.01).

The tertiary structure modeling showed that, like human C3a, grass carp C3a.1 also consists of four α-helixes, with the C-terminal region forming a long α-helix (**Figure 3B, C**). In human, the biological activity of C3a is accomplished by the C-terminal α-helix region (underlined in **Figure 3B**), thus, we wondered if the phagocytosis-stimulating activity of grass carp C3a.1 was also accomplished by the C-terminal α-helix region. To clarify this, the synthesized C3a.1-CP was used to stimulate grass carp IgM^+^ B cells. Interestingly, the results showed that, compared with the blank control, C3a.1-CP significantly enhanced the phagocytic activity of grass carp IgM^+^ B cells in a concentration-dependent manner (**Figure 6**). Like GST-C3a.1, the optimal phagocytosis-stimulating concentration of C3a.1-CP is about 10 nM, with the phagocytic activity of grass carp IgM^+^ B cells being increased by about 40%. Notably, in contrast to C3a.1-CP, huC4a-CP from human C4a inhibited the phagocytic activity of grass carp IgM^+^ B cells, indicating that the phagocytosis-stimulating activity of grass carp C3a.1-CP is specific.

### C3aR is Highly Expressed by Grass Carp IgM^+^ B Cells

**Figure 7:**
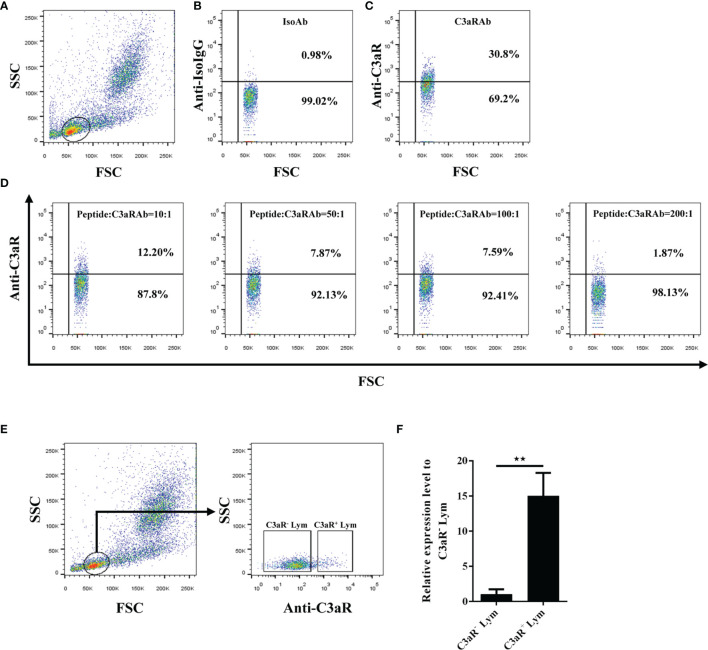
Detection of the specificity of the rabbit anti-C3aR pAbs. **(A–D)** Blocking of the anti-C3aR pAbs by preincubation with the antigenic peptide used for pAb development. **(A)** Gating strategy for flow cytometry analysis of HKLs stained with rabbit anti-C3aR pAbs. The lymphocytes (Lym) were gated to further analyze the C3aR positive cells. **(B)** Lym in HKLs stained with rabbit IgG isotype control Abs. **(C)** Lym in HKLs stained with rabbit anti-C3aR pAbs. **(D)** Lym in HKLs stained with peptide-preincubated rabbit anti-C3aR pAbs. The rabbit anti-C3aR pAbs were preincubated with the peptide at different molar ratios for 30 min, then stained HKLs for flow cytometry. **(E, F)** Expression of C3aR in C3aR^+^ Lym and C3aR^-^ Lym. **(E)** Sorting of C3aR^+^ Lym and C3aR^-^ Lym by FACS. HKLs were stained with rabbit anti-C3aR pAbs, then C3aR^+^ Lym and C3aR^-^ Lym were sorted and subjected to RNA isolation and cDNA synthesis. **(F)** The mRNA expression level of *C3aR* in C3aR^+^ Lym and C3aR^-^ Lym. The mRNA expression level of *C3aR* in C3aR^+^ Lym and C3aR^-^ Lym was analyzed by qPCR and normalized against the expression of *β-actin* using the 2^−ΔCt^ method. One representative result was shown in **(A–E)**. Data in **(F)** are presented as mean ± SEM (n = 5 fish), and the statistic *p* value was calculated by one-way ANOVA with a Dunnett *post hoc* test (**p < 0.01).

**Figure 8:**
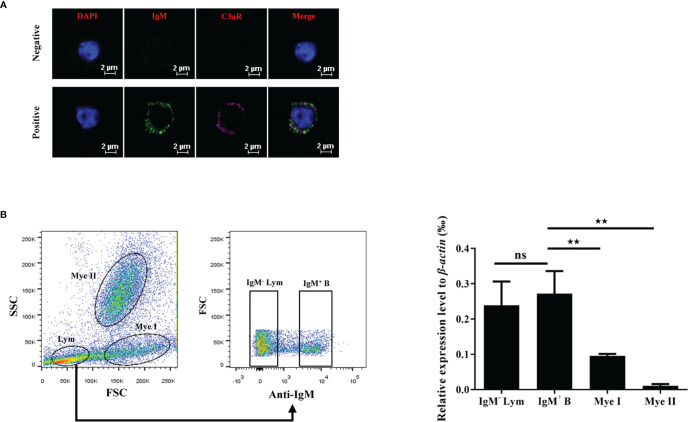
C3aR is highly expressed by grass carp IgM^+^ B cells. **(A)** Immunofluorescence microscopy of C3aR on IgM^+^ B cells. HKLs were first co-stained with mouse anti-grass carp IgM mAbs and rabbit anti-grass carp C3aR pAbs, then co-stained with Alex Flour 488-conjugated goat anti-mouse IgG (green for IgM) and Cy5-conjugated goat anti-rabbit IgG (magenta for C3aR). The mouse IgG isotype Abs and rabbit IgG isotype Abs were used as the negative controls. Nuclei were stained with DAPI (blue). One representative result was shown in the immunofluorescence images. Scale bars, 2 μm. **(B)** The mRNA expression pattern of *C3aR* in IgM^+^ B cells, IgM^-^ lymphocytes (Lym), subset I myeloid cells (Mye I), and subset II myeloid cells (Mye II). HKLs were stained with mouse anti-grass carp IgM mAbs, then cell populations including IgM^+^ B cells, IgM^-^ Lym, Mye I, and Mye II were sorted and subjected to RNA isolation and cDNA synthesis. The mRNA expression level of *C3aR* in the cell populations was analyzed by qPCR and normalized against the expression of *β-actin* using the 2^−ΔCt^ method. One representative result was shown in the dot plot of flow cytometry. Data are presented as mean ± SEM (n = 4 fish), and the statistic *p* value was calculated by one-way ANOVA with a Dunnett *post hoc* test (ns, not significant; **p < 0.01).

To further clarify the mechanism of the phagocytosis-stimulating activity of grass carp C3a.1, we detected whether grass carp IgM^+^ B cells express C3aR (**Supplementary Figure 1**). The rabbit anti-grass carp C3aR pAbs were produced, which were confirmed to be specific for C3aR for two reasons (**Figure 7**): 1) the peptide used for immunization can block the binding of anti-grass carp C3aR pAbs to lymphocytes; 2) the mRNA of *C3aR* is highly expressed in FACS sorted C3aR^+^ Lym, when compared with the C3aR^-^ Lym. After confirming the specificity of the rabbit anti-grass carp C3aR pAbs, a double staining immunofluorescence assay using rabbit anti-grass carp C3aR pAbs and mouse anti-grass carp IgM mAbs was conducted. The results revealed that grass carp IgM^+^ B cells express C3aR on the cell surface (**Figure 8A**). To confirm this result, grass carp leukocyte populations, including IgM^+^ B cells, IgM^-^ Lym, Mye I, and Mye II, were sorted from head kidney by FACS. The results of qPCR further showed that, *C3aR* was highly transcribed in IgM^+^ B cells, which was higher than that in IgM^-^ Lym, Mye I, and Mye II populations (**Figure 8B**).

### C3a.1 Enhances the Phagocytic Activity of Grass Carp IgM^+^ B Cells Through C3aR

**Figure 9:**
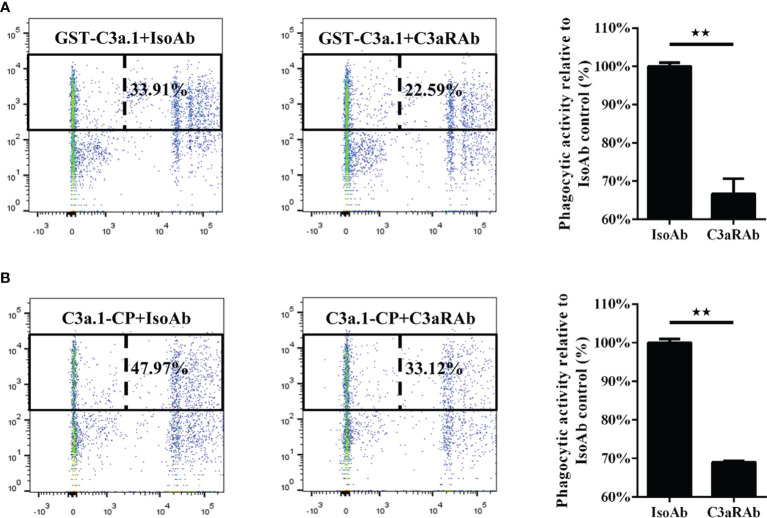
Anti-C3aR pAbs inhibits the phagocytosis-stimulating activity of C3a.1 to grass carp IgM^+^ B cells. **(A)** Anti-C3aR pAbs inhibits the phagocytosis-stimulating activity of GST-C3a.1 to grass carp IgM^+^ B cells. **(B)** Anti-C3aR pAbs inhibits the phagocytosis-stimulating activity of C3a.1-CP to grass carp IgM^+^ B cells. HKLs were incubated with GST-C3a.1 or C3a.1-CP, anti-C3aR pAbs or isotype control Abs, as well as fluorescent beads at 28°C for 2 h. Thereafter, the non-ingested beads in cell suspensions were removed, and the cells were stained with mouse anti-grass carp IgM mAbs. Finally, the phagocytic activity of grass carp IgM^+^ B cells was detected using flow cytometry. One representative result was shown in the dot plot of flow cytometry. Data are presented as mean ± SEM (n = 3 fish), and the statistic *p* value was calculated by one-way ANOVA with a Dunnett *post hoc* test (**p < 0.01).

To illustrate the role of C3aR in the phagocytosis-stimulating activity of C3a.1 to grass carp IgM^+^ B cells, a blocking experiment to C3aR was performed using anti-C3aR pAbs. The results showed that, after the C3aR on IgM^+^ B cells was blocked by the anti-C3aR pAbs, the phagocytosis-stimulating activities of both GST-C3a.1 and C3a.1-CP to IgM^+^ B cells were significantly inhibited (**Figure 9**). These results strongly demonstrated that C3a.1 enhances the phagocytic activity of grass carp IgM^+^ B cells through its receptor C3aR.

## Discussion

Unlike tetrapods with a single *C3* gene, all teleost fish possess multiple *C3* genes, such as zebrafish with eight *C3* genes (13), common carp with at least five *C3* genes (14), while rainbow trout with at least three *C3* genes (33). However, until now, the protein expression of the multiple *C3* genes in teleost fish has only be detected in rainbow trout (33). The results showed that C3.1, C3.3, and C3.4 in rainbow trout serum were 1.5-2 mg/ml, 0.3-0.4 mg/ml, and 0.3-0.4 mg/ml, respectively. This may suggest that there is dominant C3 molecule among the divergent forms of C3 in teleost fish. Interestingly, our study supports this speculation. Though a total of nine *C3* genes were discovered in grass carp, the *C3.1* was the most expressed *C3* gene at both the mRNA and protein levels. More importantly, we demonstrated that native C3a.1 was generated in the complement-activated grass carp serum. This laid an important foundation for clarifying the function of grass carp C3a.

In rainbow trout, a gel filtration fraction containing ~8 kDa molecules from complement-activated serum was found to greatly enhance particle uptake in HKLs (28). This particle-uptake enhancing fraction (PUEF-8) contained C3a, C4a, and C5a molecules. However, until now, the molecular mechanism of the PUEF-8-stimulated phagocytosis is still unknown. Interestingly, in this study, we found that C3a.1 could significantly increase the phagocytic activity of grass carp IgM^+^ B cells. Blocking experiment further revealed that this phagocytosis-stimulating activity was C3aR dependent. More importantly, we found that the C-terminal α-helix peptide of C3a.1 (C3a.1-CP) also could significantly enhance the phagocytic activity of grass carp IgM^+^ B cells. Surprisingly, the phagocytosis-stimulating activity of C3a.1-CP was higher than that of GST-C3a.1, this may because C3a.1-CP was chemically synthesized and therefore purer than the recombinantly expressed C3a.1.

Previous study revealed that rainbow trout C3a molecules could strongly stimulate the respiratory burst of HKLs in a dose-dependent manner (34). Here we show that, like C3a in mammals (11), teleost C3a are also pleiotropic molecules with multiple immune regulatory functions. In a previous study, we found that C5a could act as a molecular adjuvant in rainbow trout by enhancing Ab response to a soluble antigen (29). Thus, we wonder if C3a also is a promising vaccine adjuvant in teleost fish, which will be clarified in our future study. Nevertheless, due to the three pairs of intramolecular disulfide bonds, it was hard to express and purify recombinant C3a in the soluble form. Encouragingly, we found that the C-terminal α-helix peptide possessed the intact function of C3a, suggesting that we can use the C-terminal peptide but not the whole C3a molecule in the future study and application.

It is noteworthy that the expression pattern of C3aR on various immune cells are totally different in mammals and teleost fish. For example, in human peripheral blood, C3aR is mainly expressed on granulocytes and monocytes, but not on B cells, suggesting that granulocytes are the primary effector cells of C3a in human (27). However, in rainbow trout, C3aR is highly expressed on all IgM^+^ B cells and, to a lesser extent, all granulocytes (26). Here we also found that C3aR was highly expressed on IgM^+^ B cells in grass carp, indicating that IgM^+^ B cells acting as the primary effector cells of C3a is common in teleost fish. Interestingly, rainbow trout C3aR was found to lack a significant portion of the second extracellular loop (ECL2) when compared to that of the mammalian C3aRs (26). Here we found that the ECL2 of grass carp C3aR as well as other representative teleost C3aRs is as small as that of rainbow trout (**Supplementary Figure 1B**), indicating that the small ECL2 is common in teleost C3aR.

Phagocytosis is one of the most important defense strategies in the innate immunity of vertebrates, as well as in the initiation of the adaptive immunity (15, 16). Compared with higher vertebrates, teleost fish depend more heavily on their innate immunity to defend against various pathogens in the aquatic environment (35). Thus, phagocytosis is particularly important for the health of teleost fish. In higher vertebrates, phagocytosis is mainly conducted by professional phagocytes, including macrophages, monocytes, and granulocytes (36, 37). However, B cells in teleost fish were discovered to possess potent phagocytic, microbicidal, and antigen-presenting activities like professional phagocytes (19, 38, 39). In our previous study, we found that cathelicidin antimicrobial peptides could increase the phagocytic activity of rainbow trout IgM^+^ and IgT^+^ B cells (22). Moreover, several other molecules were also proved to up-regulate the phagocytic activity of teleost B cells, including IL-10 (23) and type I interferon-3 (25) in Japanese flounder (*Paralichthys olivaceus*), as well as chemokine CK9 in rainbow trout (24). Notably, in teleost fish, the concentration of B cells in peripheral blood is significantly higher than that in mammals. For example, B cells in the peripheral blood of rainbow trout are 5,000–15,000 cells/µL, whereas B cells in that of humans and mice are 150–720 and 1,200–3,000 cells/µL, respectively (19). The large number of phagocytic B cells in teleost fish makes them an ideal target for developing effective vaccines, adjuvants, or immunopotentiators in fish farming (39). Thus, discovering molecules like C3a with phagocytosis-stimulating activities to teleost B cells is important not only for fish immunology, but also for preventing fish diseases.

Taken together, this study clearly illustrated the cross talk between C3a and phagocytic B cells in teleost fish, uncovered the novel phagocytosis-stimulating activity of teleost C3a, and laid an important foundation for further study the application potential of C3a or C3a derived C-terminal peptide as immunostimulants or as molecular adjuvants by targeting B cells.

## Data Availability Statement

The datasets presented in this study can be found in online repositories. The names of the repository/repositories and accession number(s) can be found in the article/**Supplementary Material**.

## Ethics Statement

The animal study was reviewed and approved by Ethical Committee of Huazhong Agricultural University.

## Author Contributions

Z-YM performed most of the experiments, analyzed most of the data, and wrote the preliminary manuscript. J-XL helped with most of the experiments, especially the phagocytosis assay. W-SL helped with some of the experiments, especially the Western blot experiments. YS helped with some of the experiments, especially the blocking experiment. C-SW searched the C3 genes in the grass carp genome. Y-ZH helped with reagent preparation and data analysis. JL helped with experiment design. Y-AZ helped with experiment design and revised the manuscript. X-JZ designed the research, analyzed some of the data, and revised the manuscript. All authors contributed to the article and approved the submitted version.

## Funding

This work was supported by the National Natural Science Foundation of China (31972824 and 31602184), the Open Project of Guangdong Provincial Key Laboratory of Pathogenic Biology and Epidemiology for Aquatic Economic Animals (PBEA2021ZD02), the National Key Research and Development Program of China (2018YFD0900505), and the Fundamental Research Funds for the Central Universities (26 62018QD053).

## Conflict of Interest

The authors declare that the research was conducted in the absence of any commercial or financial relationships that could be construed as a potential conflict of interest.

## Publisher’s Note

All claims expressed in this article are solely those of the authors and do not necessarily represent those of their affiliated organizations, or those of the publisher, the editors and the reviewers. Any product that may be evaluated in this article, or claim that may be made by its manufacturer, is not guaranteed or endorsed by the publisher.
