# Supplementary material for: Complement C3a Enhances the Phagocytic Activity of B Cells Through C3aR in a Fish
Source: Front Immunol. 2022 Mar 21;13:873982. doi: 10.3389/fimmu.2022.873982 (PMC8977587; doi:10.3389/fimmu.2022.873982)
Supplement: Supplementary file 1 [file DataSheet_1.docx]

Supplementary Material

# Supplementary Table

| **Supplementary Table I.** Primers used in this study. | | |
| --- | --- | --- |
| Name | Sequence (5’ to 3’) | Application |
| β-actin-QF | AGCCATCCTTCTTGGGTATG | Quantitative real-time PCR |
| β-actin-QR | GGTGGGGCGATGATCTTGAT |  |
| C3.1-QF | GAGAATAAACTCGGCTACACCT |  |
| C3.1-QR | ATCATCTCCTCCTCTTCTGTCT |  |
| C3.2-QF | GGATAAAGCCTGTGAAGATGAC |  |
| C3.2-QR | TCAACAGTCCCTCAACGCAAT |  |
| C3.3-QF | ATGACGGCATTTGTGCTCATTG |  |
| C3.3-QR | GCTACCGTCCAGACTTGAGAT |  |
| C3.4-QF | GAAGCACTTAGAAGGCGAACA |  |
| C3.4-QR | CCTCTAAACGGTTGAAGGTCA |  |
| C3.5-QF | CCTGGTGATAGCGAAGGATTT |  |
| C3.5-QR | GTGCCATAACCTCCGTAGCG |  |
| C3.6-QF | GAAAAACGCATAACAGATGGACT |  |
| C3.6-QR | CTTCAGGCAGAGCATAATACAG |  |
| C3.7-QF | CTGTAGCCAAAGAGGGCAAGT |  |
| C3.7-QR | AGTGACATTGAAGGCTGGGAG |  |
| C3.8-QF | AGGCTCACTCAGTTTAGGACC |  |
| C3.8-QR | GAAAGATGGCATTATCCACAGC |  |
| C3.9-QF | GAGGAGGAAGATGAGGATTTAG |  |
| C3.9-QR | AACTGTCAGGAAGAACGGTTTC |  |
| C3aR-QF | CATTTCTCCTCGGAGTTCCTG |  |
| C3aR-QR | GAGGTCGGCTGTTGCTAGATT |  |
| exC3a.1-F | CGGGATCCGCTGAGAGTCTCCTGCAGAT | Prokaryotic expression |
| exC3a.1-R | CCGCTCGAGTTAGCGAGCCAGAATCATCTCCT |  |

The restriction enzyme sites are underlined

# Supplementary Figure

**
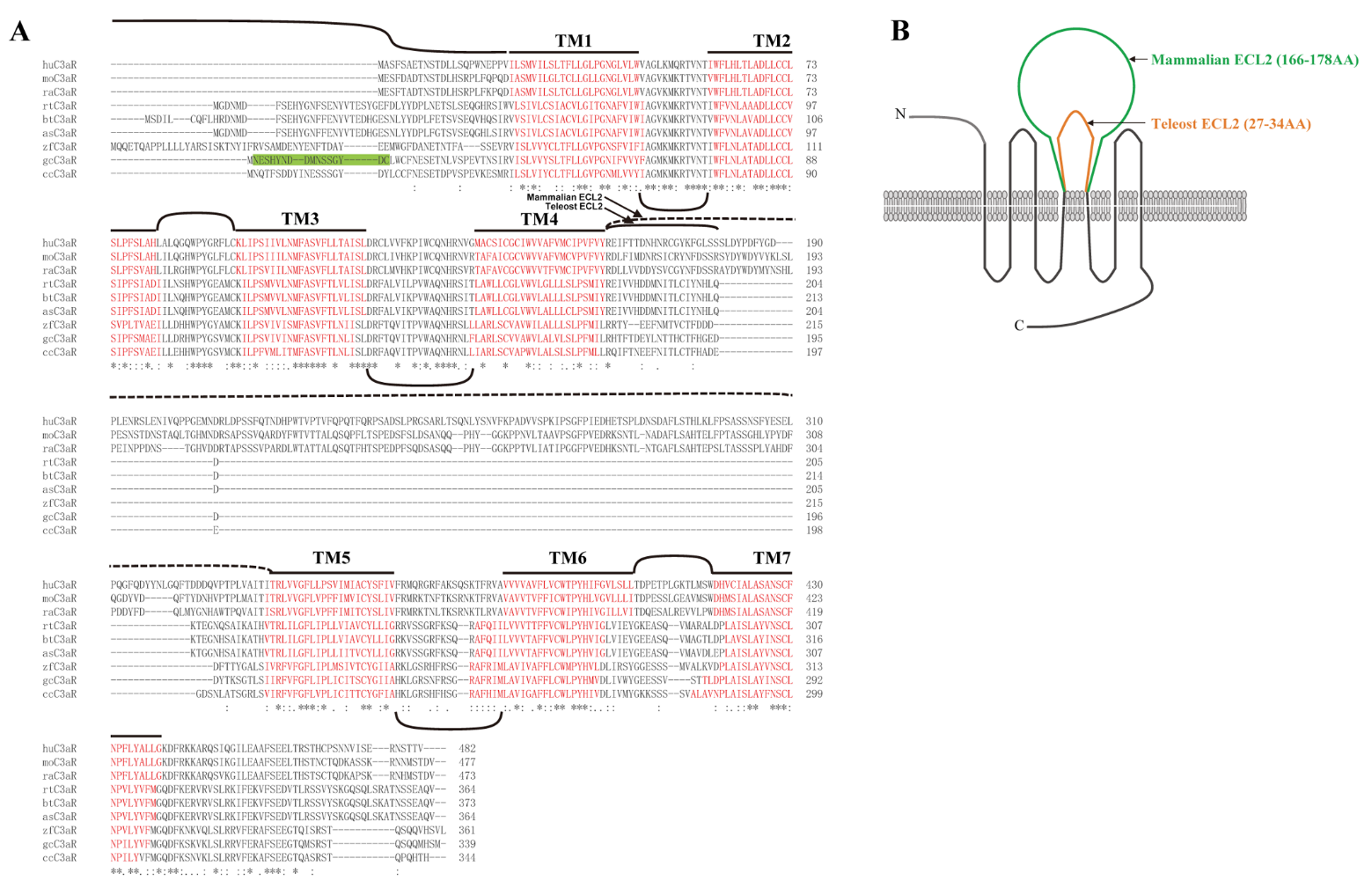
**

**Supplementary Figure 1.** The amino acid sequence and domain structure of grass carp C3aR. **(A)** Amino acid sequence alignment of grass carp C3aR with C3aRs from other representative vertebrates, including human (hu; *Homo sapiens*), mouse (mo; *Mus musculus*), rat (ra; *Rattus norvegicus*), rainbow trout (rt; *Oncorhynchus mykiss*), brown trout (bt; *Salmo trutta*), Atlantic salmon (as; *Salmo salar*), zebrafish (zf; *Danio rerio*), and common carp (cc; *Cyprinus carpio*). GenBank accession numbers of the used sequences are shown below: rtC3aR, NM_001124403.2; gcC3aR, MG599686.1; zfC3aR, P0C7U4; ccC3aR, XP_018919368.2; btC3aR, XP_029546122.1; asC3aR, XP_013994139.1; huC3aR, NP_001313404.1; moC3aR, AAH03728; raC3aR, O55197. The transmembrane regions (TM1-7) were predicted by the TMHMM program (<https://services.healthtech.dtu.dk/service.php?TMHMM-2.0>) and are shown in red. The extracellular and intracellular domains are denoted by upward and downward arc symbols, respectively. The N termini are indicated by a line linked to TM1. Asterisks, colons, and single dots denote the identities of the corresponding amino acid residues, while dashes denote gaps. The peptide sequence at the N-terminal of gcC3aR used to generate pAbs is shaded green. **(B)** Schematic structure of vertebrate C3aRs. The second extracellular loops (ECL2) of the C3aRs between TM4 and TM5 are shown. Numbers in brackets are the number of amino acid residues of the corresponding ECL2.
